# Determining the feasibility of randomising infants, children and young people to invasive and non-invasive urine sampling techniques (FROG): Protocol for a multicentre randomised controlled feasibility trial and mixed methods perspectives’ study of RCT feasibility.

**DOI:** 10.3310/nihropenres.14114.1

**Published:** 2025-11-21

**Authors:** Paula Taylor Miller, Clíona McDowell, Ashley Agus, Lynn Murphy, Stuart Hartshorn, Srinivas Bandi, Bronagh Blackwood, Nefyn Williams, Damian Roland, Kathryn Ferris, Andrew Marshall, Alastair Sutcliffe, Mike Clarke, Kerry Woolfall, Thomas Waterfield

**Affiliations:** 1 Northern Ireland Clinical Trials Unit, Belfast Health and Social Care Trust, Belfast, Northern Ireland, UK; 2 Birmingham Women's and Children's NHS Foundation Trust, Birmingham, England, UK; 3 Paediatric Emergency Medicine Leicester Academic (PEMLA) Group, Children’s Emergency Department, Leicester Royal Infirmary, Leicester, UK; 4 Wellcome-Wolfson Institute for Experimental Medicine, Queens University Belfast, Northern Ireland, BT9 7BL, UK; 5 University of Liverpool Institute of Population Health, Liverpool, England, UK; 6 SAPPHIRE Group, Population Health Sciences, Leicester University, Leicester, UK; 7 Oxford University Hospitals NHS Foundation Trust, Oxford, England, UK; 8 University College London, Great Ormond Street Institute of Child Health, London, England, UK; 9 School of Medicine, Dentistry and Biomedical Sciences, Centre for Public Health, Queens University Belfast, Northern Ireland, UK

**Keywords:** Paediatric, urinary tract infection, suprapubic aspirate, catheter, clean- catch, feasibility, RCT

## Abstract

**Background:**

Urinary tract infections (UTI) are the second most common serious bacterial infection in children. When healthcare practitioners are unsure if an infant, child, or young person has a UTI they perform a urine test. A midstream sample is recommended by the National Institute for Health and Care Excellence (NICE) for obtaining urine for testing. However, collecting urine from children who are unable to provide a midstream urine sample is challenging. Samples can be collected either by non-invasive (clean catch) or invasive methods (trans-urethral bladder catheter or suprapubic aspirate). Non-invasive methods are slow and prone to contamination but are painless. Invasive methods are less prone to contamination and are quick but can cause pain and distress.

**Methods:**

This is a mixed methods feasibility study comprising of three parts. Part 1 is a pragmatic multicentre randomised controlled feasibility trial. The trial aims to evaluate the feasibility of conducting an RCT comparing invasive and non-invasive sampling methods for infants, children and young people (N = 100). Resource use will be assessed by parent reported questionnaire and mixed methods descriptors reported by healthcare professionals (n = 24). A cost analysis will assess the urine collection methods, informing a future cost-effectiveness analysis. Part 2 is an embedded mixed methods perspectives’ study including interviews with parents (n = 15-20) and children (n = 10-15), five focus groups (n = 6-8 per group) and interviews (n = 10) with healthcare professionals aiming to assess feasibility and acceptability of the trial. Part 3 is a stakeholder (n = 40) consensus meeting determining a final definitive study design.

**Discussion:**

The results of the study will inform a recommendation and decision on progression and design of a definitive RCT for infants, children and young people who have a suspected urine infection but cannot provide a midstream urine sample.

**Trial registration:**

International Standard Randomised Controlled Trial Number (ISRCTN) 84676764: Feasibility of conducting a randomised controlled trial (RCT) comparing invasive and non-invasive urine sampling techniques in children under 16 years old with a suspected urinary tract infection ^1^.

## Plain Language summary

Internationally, the approach to urine collection varies. In Europe and North America, national guidelines typically favour invasive urine collection methods, given their advantage of much lower rates of bacterial contamination. However, invasive urine collection methods are associated with the disadvantages of pain and small risk of trauma. In the UK, the National Institute of Health Research (NICE) recommend administering invasive methods only if it is not practical or possible to collect urine by non-invasive urine sampling methods. Instead, a "mid-stream" or "clean catch" non-invasive method is recommended by NICE as a first line method of urine collection.

Infants, children and young people who cannot provide a mid-stream urine sample by collecting their urine in a pot while they go to the toilet, may be supported by parents/ guardians and healthcare professionals, when collecting the child's urine. This is called the clean catch method of urine collection. This method is associated with a higher rate of contaminated urine samples and can be a distressing and lengthy process, especially in time critical emergency care settings. A UK-based study is required to determine which invasive or non-invasive urine sampling infants, children, and young people should be offered across hospital settings.

A randomised controlled trial (RCT) is considered to provide the strongest evidence when comparing the various urine collection methods, but it is not clear if potential participants could be recruited to such a study. This current feasibility study is required to determine if a definitive RCT would be possible and, if so, to inform its design.

The researchers aim to conduct a study of feasibility to assess which participants and interventions should be included in a subsequent randomised controlled trial, explore potential barriers to recruitment and determine the feasibility of randomisation to invasive versus non-invasive urine testing.

**Protocol**

Version 3.0 24 April 2025

## Background

Urinary tract infections (UTIs) are the second most common serious bacterial infection in children, accounting for a substantial number of presentations to both primary and secondary care ^2^. By age 16, approximately 1 in 10 girls and 1 in 30 boys will have experienced a UTI ^3, 4^. Across childhood, the prevalence of UTI is estimated to be around 5% ^5– 7^, with symptoms often non-specific, including fever, vomiting, abdominal pain, and lethargy ^8, 9^.

Urine testing is routinely performed when a UTI is suspected, guiding antibiotic treatment and follow-up. Prompt diagnosis and management are essential to prevent complications such as severe infection and renal scarring ^10^. NICE guidance (NG224, 2022) recommends clean catch urine (CCU) collection where feasible, discouraging invasive methods such as transurethral bladder catheterisation (TUBC) or suprapubic aspiration (SPA) unless non-invasive methods are impractical ^8^.

Non-invasive methods are often preferred due to their painless nature and suitability for primary care. However, CCU is associated with high contamination rates and is time-consuming. Three UK studies involving 1,093 children reported contamination rates of 26–36% for CCU samples ^9– 12^, compared to 12% for TUBC and 1% for SPA ^9^. Contaminated samples contribute to poor antimicrobial stewardship and increased antimicrobial resistance (AMR), which has been identified by the World Health Organisation as a major global threat ^13^.

E. coli is the predominant pathogen in paediatric UTIs. Resistance has increased, with 30% of E. coli UTIs in England resistant to Trimethoprim and 10% to Cefalexin ^8, 13^. Reducing unnecessary antibiotic use is key to combating AMR. However, reliance on contaminated non-invasive samples increases false positives, leading to overtreatment, unnecessary investigations, hospital admissions, and distress for families.

Internationally, approaches to urine collection vary. In Europe and North America, national guidelines typically favour invasive methods ^9– 12^ due to their significantly lower rates of bacterial contamination ^14– 18^. Transurethral bladder catheterisation (TUBC) and ultrasonography-guided suprapubic aspiration (SPA) are generally regarded as low-risk procedures in the US, Europe, the Middle East, and Africa when performed by trained professionals ^19, 20^. Despite this, there is limited research exploring the views of parents/guardians and healthcare professionals (HCPs) on invasive sampling, even though such techniques have historically been described as emotionally and physically traumatic for infants and families ^19, 21, 22^.

Recent UK data suggest that both HCPs and parents consider invasive methods acceptable in febrile or seriously unwell children when a urine sample is urgently required ^23^. Clean catch urine sampling, considered the gold standard non-invasive method by UK HCPs, avoids painful procedures but can be distressing for infants and stressful for parents managing the process ^21, 23^. These challenges may be heightened in time-critical emergency care settings.

Despite the clinical importance of urine sampling, there is limited evidence regarding the acceptability and feasibility of comparing invasive and non-invasive methods in a UK context. A feasibility study is therefore required to determine whether a definitive randomised controlled trial (RCT) comparing these approaches can be conducted, and to inform its design.

## Design

**Figure 1.**
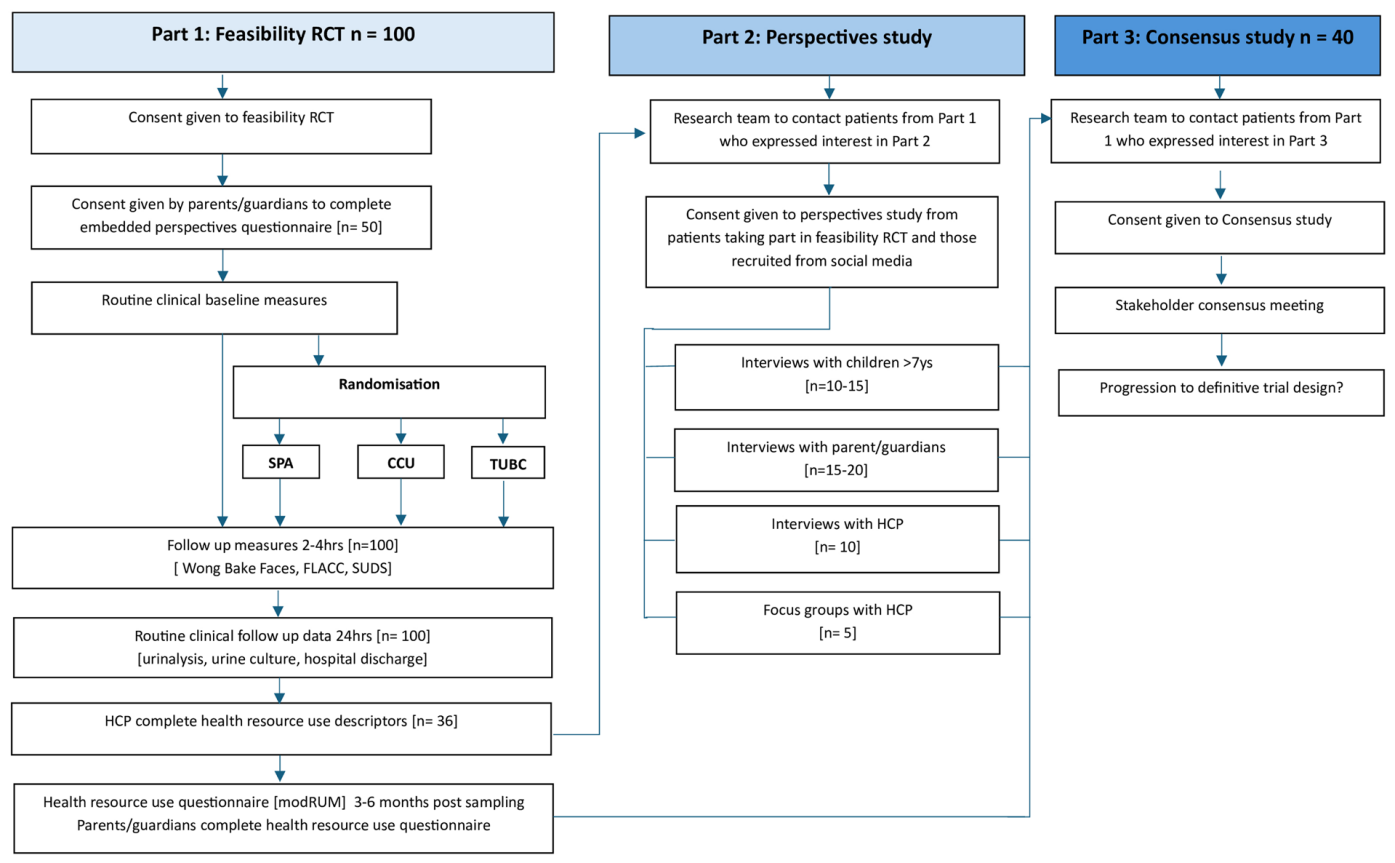
Study design phases; Parts 1, 2 and 3.

**Figure 2.**
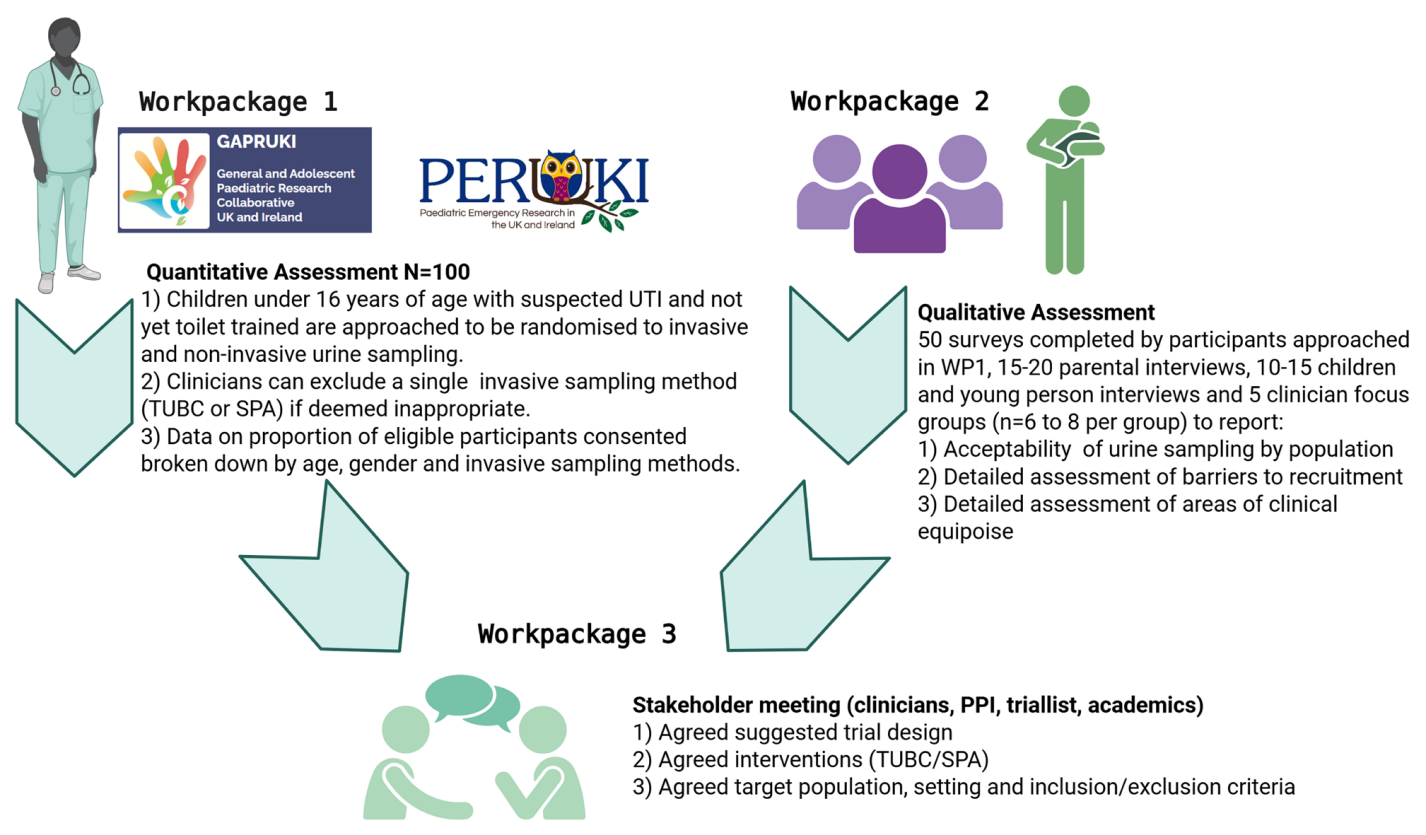
Flow diagram of the FROG mixed methods feasibility study involving three linked parts. Work Package 1 (Part 1) is a randomised controlled feasibility trial, Work Package 2 (Part 2) is a perspectives study, followed consecutively by Work Package 3 (Part 3) stakeholder consensus meeting.

This is a mixed methods feasibility study including three parts outlined in Figure 1 and Figure 2.

### Aim

To conduct a study of feasibility to assess which participants and interventions should be included in a subsequent RCT, explore potential barriers to recruitment and determine the feasibility of randomisation to invasive versus non-invasive urine testing.

### Objectives

1. To determine the number of potential participants with suspected UTI presenting to a range of clinical settings, including emergency care, inpatients, and outpatients.
2. To conduct a quantitative assessment of the ability to screen, recruit and randomise children and young people to one of three interventions (CCU, SPA and TUBC).
3. To explore the views of parents, children, young people, and clinicians on the acceptability of different collection methods, and the appropriate population for inclusion in a future study.
4. To identify potential barriers to recruitment and consent.
5. To establish the most appropriate design, including important patient centred outcomes, for use in a future study.
6. To perform a cost analysis of the three urine collection methods to inform the resource planning and design of a future cost-effectiveness analysis.

## Methods

### Patient and Public Involvement

Patient and Public Involvement (PPI) groups in Northern Ireland, Liverpool (Generation R) and via the GAPRUKI network contributed to the preparation of the study design and outcomes. A total of eighty individuals including children, young people, and adults were involved through a mixture of virtual meetings (n=3) and surveys (n=2) prior to the study grant application.

PPI activity for the FROG study will include liaison with PPI groups from charitable organisations and primary schools. A PPI competition will be held for children to design a study logo and develop the trial identity which will then be created by professional graphic designers. The PPI group will contribute to the development of all participant information resources, the interpretation of results, report writing and dissemination of study results and findings. A PPI representative will be invited to participate in Trial Management Group meetings and there will be a PPI representative on the Trial Steering Committee. PPI representatives will be invited to participate in other relevant meetings and the consensus meeting to ensure that the research is relevant to patients. PPI members will be trained and supported for this role.

All members of the PPI group and PPI representatives will receive reimbursement of expenses, in line with NIHR Centre for Engagement and Dissemination recommendations. PPI representatives involved in the study management groups will be acknowledged for their contributions. The Guidance for Reporting Involvement of Patients and the Public, Version 2 (GRIPP2 checklists) ^24^ will be used for reporting on patient and public involvement (PPI) in the publication reporting study results.

## Part 1

**Figure 3.**
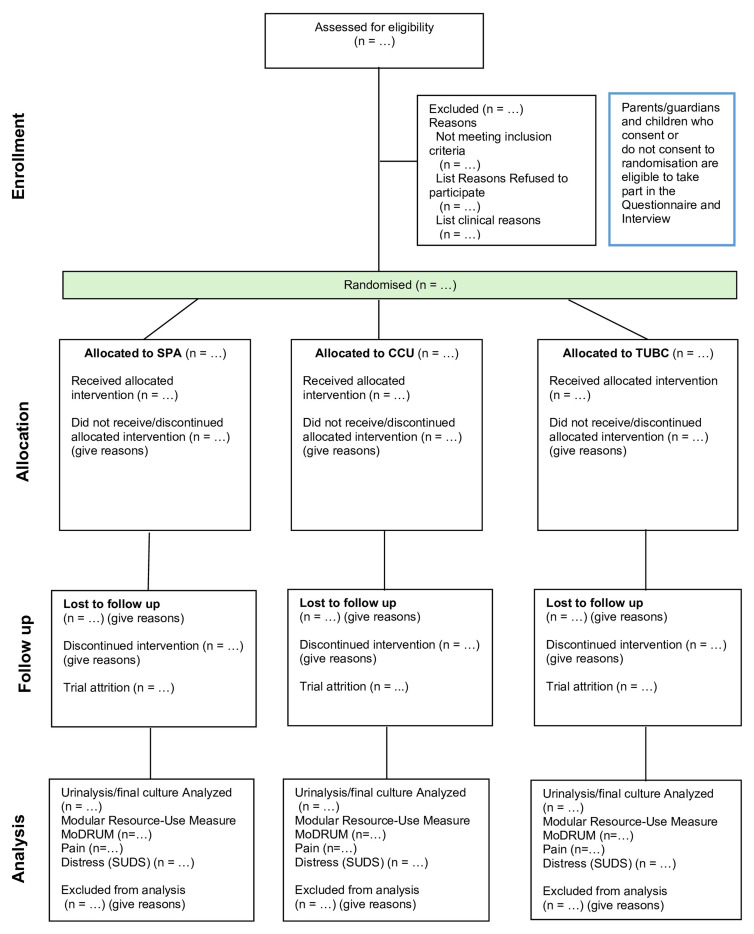
CONSORT diagram depicting participant flow through Part 1 (randomised feasibility trial).

**Figure 4.**
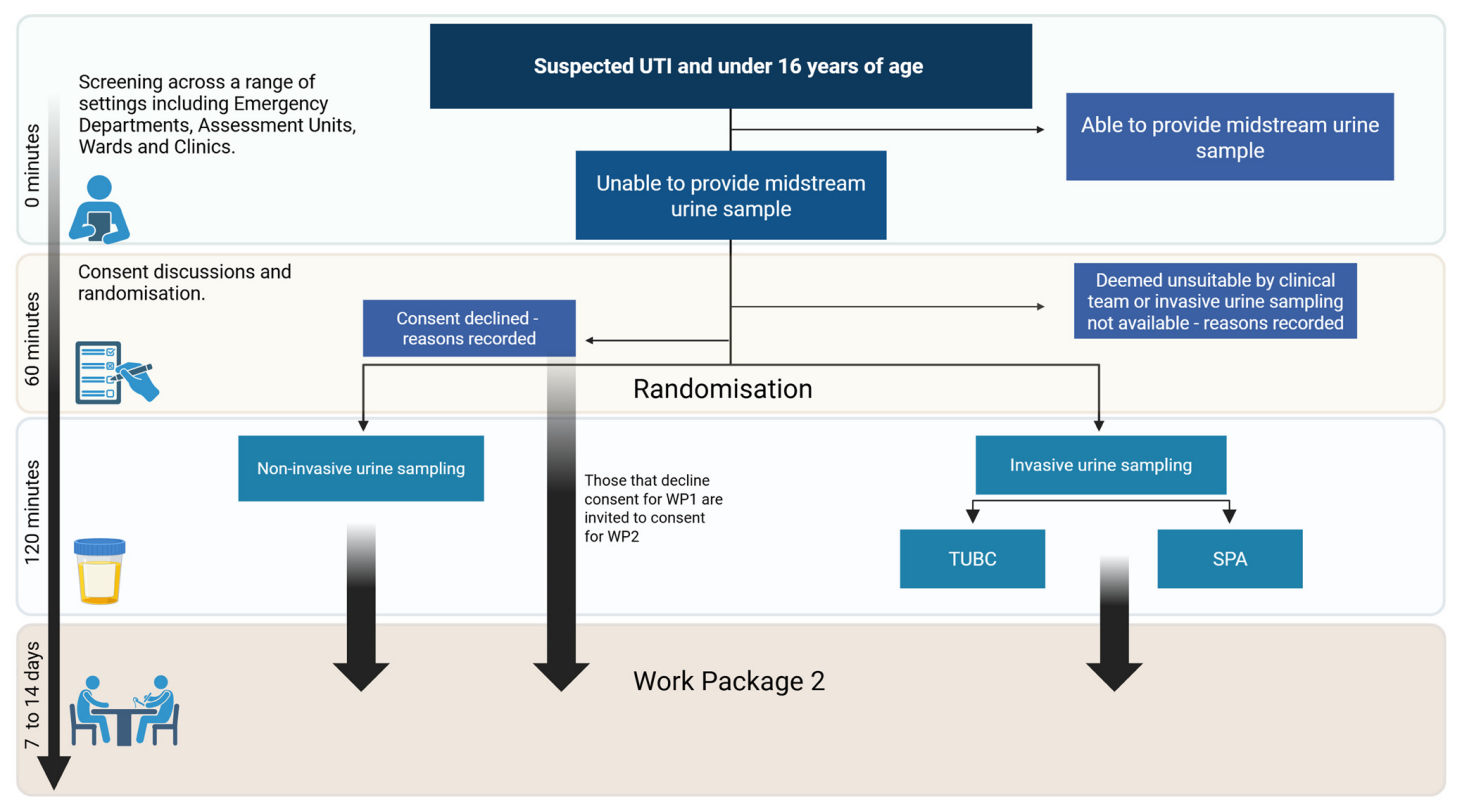
Pictorial representation of participant journey though the randomised feasibility trial (Part 1).

A pragmatic multicentre randomised controlled feasibility trial (n = 100) will assess the feasibility of randomising children to invasive and non-invasive urine sampling. The CONSORT ^25^ diagram depicting participant flow through the randomisation element of the feasibility trial is presented in Figure 3 and pictorial representation in Figure 4.

### Primary outcome

The primary outcome is the proportion of participants who are offered the study for whom consent to randomisation is obtained.

### Secondary outcomes

1. Age, gender, ethnicity and basic demographic data of participants who consent

2. Proportion of presenting patients who are judged unsuitable for the study

3. Proportion of participants with consent for randomisation to CCU, TUBC or SPA

4. Proportion of participants with consent for randomisation to CCU or TUBC only

5. Proportion of participants with consent for randomisation to CCU or SPA only

6. Proportion of participants in each randomised group who received the allocated intervention

7. Rates of contamination by urine collection method

8. Safety as defined as the incidence of adverse events

9. Time to collect urine sample

10. Pain score associated with urine sampling

11. Final diagnosis of UTI

12. Resource use and costs

### Trial setting

The randomised controlled feasibility trial will be conducted at six hospital sites in the UK.

### Trial population

Participants will be screened from attendances to paediatric EDs, assessment units, inpatient wards, and outpatient clinics at recruiting sites. Neonatal units will be excluded from recruitment. Patients assessed for eligibility and reasons for exclusion will be recorded.

### Inclusion criteria

1. Child under 16 years of age at presentation.

2. Requiring urine testing for suspected UTI.

3. Cannot provide a mid-stream urine sample (are not toilet trained).

### Exclusion criteria

1. A clinical need to collect an immediate invasive urine sample without delay

2. Participants where both methods of invasive urine sampling are deemed inappropriate by the treating clinician or are unavailable.

3. Children sedated or admitted to intensive care units at the time of screening

4. Language issues (not overcome with use of translators and available translated information sheets).

5. Parent or legal representative unavailable to provide informed consent.

6. Consent declined.

### Sample size

As a feasibility study, a formal sample size calculation is not required. A target of 100 participants in Part 1 of the study is based on the need to recruit enough participants to Part 2 who both consent and decline to randomisation.

### Informed consent procedure

Informed assent (as appropriate) and consent will be obtained from children and/or parents/guardians following explanation and understanding of the study aims, objectives and processes. A member of the clinical team will first approach the parent(s)/guardian(s) of the child. If interested, they will be introduced to a research team member who will explain the study aims, procedures, and urine sampling methods. Families will be provided with a parent/guardian information leaflet and an explainer video; children will receive age-appropriate information sheets. Time will be given (approximately one hour) to consider participation and ask questions.

Written consent to join Part 1 of the study, including randomisation, will then be sought. Families who decline randomisation will receive standard care and be registered in the study system. Parents/guardians and children (over 5 years) consenting and non-consenting to randomisation in Part 1 remain eligible for Parts 2 and 3 of the study.

### Trial interventions

Eligible participants who consent to be randomised will be assigned to one of the interventions; Invasive Trans-Urethral Bladder Catheterisation (TUBC) or Invasive Suprapubic Aspiration (SPA) or Non-invasive Clean Catch Urine (CCU) ( Figure 3).

**Figure 5.**
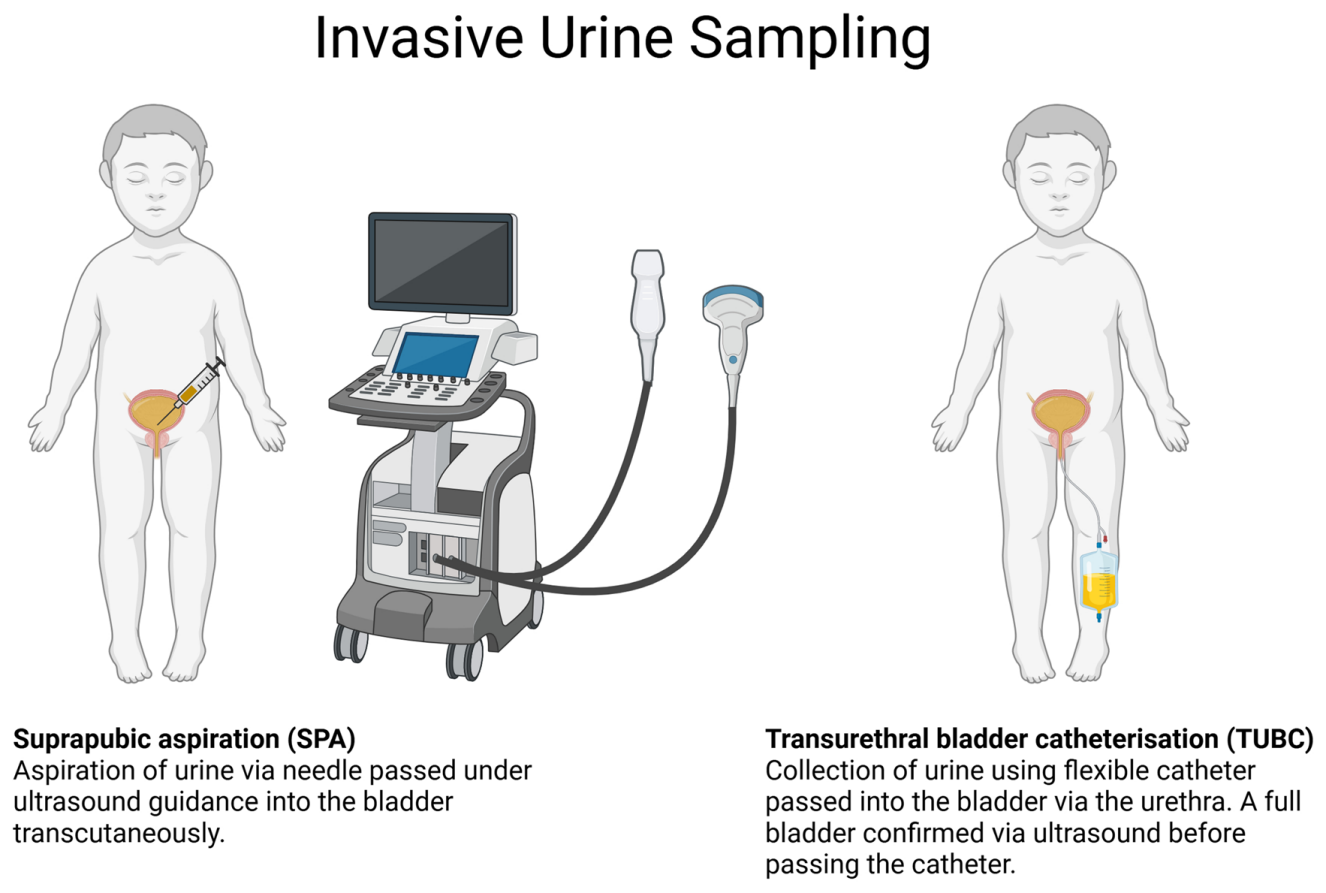
Visual summary of invasive urine sampling methods; Suprapubic Aspiration (SPA) and Transurethral Bladder Catheterisation (TUBC).

TUBC involves passing a flexible catheter into the bladder via the urethra. SPA involves placing a needle through the skin of the abdomen directly into the bladder. Non-invasive urine collection involves catching a urine sample in a small dish. Sites will follow local policies and procedures for urine sampling collection. In the event that a sampling method has been discontinued, an alternative and clinically appropriate sampling method will be administered to the child. Adherence to intervention and comparator will be recorded by discontinuation of sampling method. These procedures are not protocolised and will be performed, discontinued or modified in accordance with standard local practice. Sampling method will be discontinued at the patient’s request and guided by clinical judgement. Post-trial care will be provided as per standard local clinical practice. A full summary of all trial interventions is shown in Figure 5.

### Assignment of intervention

Participants will be recruited and randomised via an automated web-based system using randomly permuted blocks in a 1:1:1 ratio to CCU, TUBC, or SPA. If one of the invasive methods is contraindicated or unavailable, it will be excluded, and randomisation will proceed between the remaining options. Participants can be included as long as one invasive method is appropriate and available. In such cases, randomisation will occur in a 1:1 ratio between CCU and the available invasive method.

The randomisation sequence will be managed by a third-party provider and concealed from the trial statistician and those enrolling participants. Allocation will remain concealed until the point of randomisation. Blinding will not be applied thereafter, reflecting the pragmatic design focused on assessing feasibility for a future trial.

### Participant assessments

**Figure 6.**
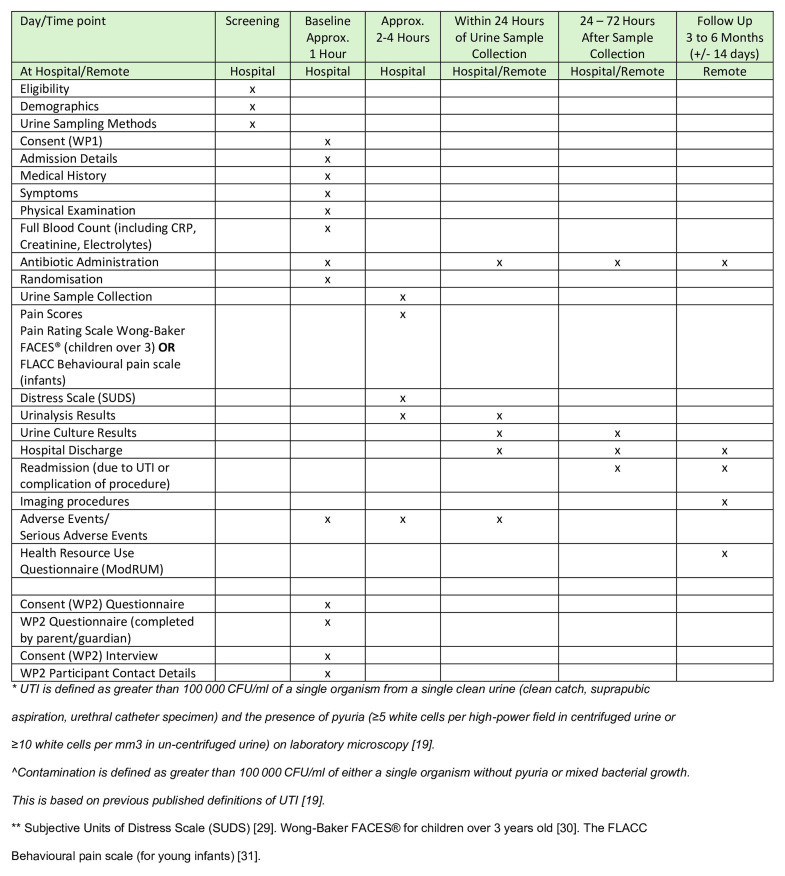
SPIRIT ^26, 27^ Schedule of enrolment, interventions and assessments.

The frequency of assessments and patient journey are detailed in Figure 4 and Figure 6.

### Data collection

Each participant will be allocated their own unique participant study number during the recruitment/randomisation process, which will be used throughout the study for participant identification on all data collection forms and questionnaires. An entry will be recorded in the patients’ medical notes noting enrolment into the study.

All data for an individual participant will be collected and recorded in source documents and transferred onto a bespoke, web-based, electronic case report form (eCRF) for the study. For routinely collected clinical data, the NHS record will be the source document. The Modular Resource-Use Measure ^28^ will be administered between 3–6 months post urine sampling method. SUDS ^29^ distress and pain scores (Wong Baker ^30^, FLACC ^31^ will be child and parent reported respectively following administration of urine sampling. Participant identification on the eCRF will be through their unique participant study number, allocated at the time of randomisation. Adverse event (AE) reporting will follow the Health Research Authority (HRA) guidelines on safety reporting in non-CTIMP studies. Available outcome data will be collected for participants who discontinue or deviate from the protocol.

### Data management and monitoring

Participant data will be entered into screening and clinical databases and processed in accordance with the Data Management Plan (DMP). Electronic data queries will be issued to site staff to clarify or complete missing information, with responses and amendments made directly within the study database. Monitoring, audits, ethics reviews, and regulatory inspections will be conducted with site agreement and access to source data and documentation. Patient safety, adverse events and serious adverse events will be routinely monitored and reported to the trial coordinating centre across host sites.

Confidentiality will be maintained in line with applicable laws and regulations. Data validation checks will identify discrepancies such as out-of-range values, inconsistencies, or protocol deviations. A Data Monitoring and Ethics Committee (DMEC) will review study data at scheduled intervals. The study will adhere to best practice principles for sharing individual participant data from publicly funded clinical trials ^32^. Host sites, Sponsor and funder will be notified of substantial and non-substantial study amendments through CPMS and the trial coordinating centre.

### Data analysis part 1

As this is a feasibility study, analysis will be descriptive in nature. We will describe baseline characteristics and outcomes using suitable measures of central tendencies; means and medians with the associated standard deviations/interquartile ranges for continuous data; and frequencies and proportions for categorical data. A sensitivity analysis will be performed to determine numbers of children perceived to be at higher risk, the reasoning as to why they were higher risk (e.g. age, fever, signs of sepsis, symptoms) and the proportion of higher and lower risk children successfully recruited and randomised. Interim analysis will be conducted including safety reporting to the independent DMEC, who provide input to final decisions made by the Trial Steering Committee (TSC).

### Health economics analysis

A detailed costing analysis will be performed of the different methods of urine collection in children up to the point of achieving a definitive sample from a hospital perspective. Resource use will be prospectively collected as will staff time (nursing and medical) and equipment for each method in a sub sample of episodes from each site. A version of the ModRUM ^28^ adapted for completion by a parent/guardian will be used to collect healthcare resource use after the participants have left the hospital setting up to a maximum of 6 months post-randomisation. Unit costs from publicly available sources (e.g. NHS Reference Costs, unit costs of health and social care) will be applied to the resource use where possible. Other sources, such as hospital costing departments and the literature, will be used when this is not possible. Costs will be presented in GBP£. Costs associated with the initial sampling will be included as will any repeat sampling and any follow up investigations to estimate the mean cost per definitive UTI diagnosis ^33^. Sensitivity analyses will be performed to explore impact on the cost estimates of variations in key parameters e.g. time estimates, staff grade.

## Part 2: Mixed methods perspectives study

An embedded mixed methods study will explore the perspectives of parents, children, young people, and healthcare practitioners about the feasibility of the proposed trial. This research will include a perspectives’ questionnaire which will be set within the randomised feasibility trial (Part 1). Interviews will be conducted with parents and children as well as focus groups and interviews with HCPs who are involved in and conducted the randomised feasibility trial (Part 1). Participants external to the feasibility trial will also be sought from social media channels enabling sample diversity. An exploration of topics will provide qualitative and quantitative insight into the acceptability of different sampling collection methods, the population for inclusion in a future study, potential barriers to recruitment and consent and important patient centred outcomes for use in a future trial.

### Part 2 Population: Parents/ guardians and children

*Inclusion criteria*

Parents/guardians of children (0 to under 16 years) and children (aged 7 to under 16 years) who are approached to participate in Part 1, including those who decline randomisation are eligible to take part in Part 2 of the study.

In addition, parents/guardians of children (0 to under 16 years) and children (aged 7 to under 16 years) who have required urine testing in hospital setting for suspected UTI in the last three years are also eligible to take part in the perspectives’ aspect of feasibility.

*Exclusion Criteria* for part 2 of the study include language issues (not overcome with use of translators and available translated information sheets) and those who declined consent to join Part 2.

### Part 2 Method: Interviews and questionnaires with parents/guardians and children

***Recruitment and sampling***

**Recruitment Route 1: Hospital Sites hosting the randomised controlled feasibility trial**

Participants, including those that decline randomisation, recruited during Part 1 will be invited to the perspectives’ study element (Part 2) and asked to complete a brief perspectives questionnaire following the study recruitment discussion. If both parents are present, both will be asked to consent and complete a questionnaire. Based on previous studies in similar settings, 50 completed questionnaires are expected.

Parents will also be invited to take part in an interview with a researcher from the University of Liverpool at a later date. Child assent and parental consent will also be sought for children to take part in an individual or joint interview with parents if the child is deemed well enough to broach the study at that point in time.

**Recruitment Route 2: Social Media**

To ensure diversity across geographic regions and ethnicities within the UK, participants will be recruited through tailored social media advertising and targeted emails to relevant UK-based charities and organisations. The research team will contact gatekeepers (e.g. charity leads/Chief Executive Officers) of support groups for parents/legal representatives whose children have required urine testing for suspected UTI in the last three years. The research team will send an age and language-appropriate Participant Information Sheet, check eligibility and whether a translator will be required for the interview. Potential participants will be asked to read the study information and ask any questions they may have before being sent a link by email to an online consent form to complete. An email or paper version can be sent on request if preferred (e.g. for children). Purposeful sampling will be conducted to ensure parents and children (aged 7 to under 16 years) reflect the various settings, ethnic diversity and representation of age ranges of infants, children and young people who would be eligible for a definitive study ^34– 36^.

### Interview procedures: Parents/guardians and children

The University of Liverpool team will contact parents and children to arrange an interview within one month of consent. Parents and children will be offered online or face to face (in the Northwest of England) interviews. All interviews requiring a translator will be conducted online via Microsoft Teams. The researcher will check whether younger children wish to be interviewed alone or with a parent present. Interviews will be conducted using the age/level of understanding appropriate interview topic guide and in line with University of Liverpool’s safeguarding policies and procedures for interviewing research participants. Consent for audio recording of the interview by Dictaphone will be checked verbally before the interview commences. The topic guide has been informed by previous feasibility studies conducted in paediatric NHS settings ^37– 39^. Respondent validation will be used so that previously unanticipated topics will be added to the topic guide and discussed with participants as interviewing and analyses progress. Interviewers will refer to the distress guide and any distress expressed by participants during the interviews will be managed with care and compassion. Participants will be free to decline to answer any questions that they do not wish to answer or to stop the interview at any point. All families will be supported in obtaining appropriate help.

Approximately 25–35 participants will be interviewed (~15–20 parents and ~10–15 children) selected from the two recruitment routes. The final number will depend on the point of information power ^40^, which considers factors including quality of data and sample variance. All families who express an interest in taking part but are not selected for an interview will be contacted via telephone or email to thank them for their interest in the study.

### Part 2: Population healthcare practitioners

*Inclusion Criteria*

NHS healthcare practitioners (doctors, nurses, research staff and Allied Health Professionals) who are or are not involved in recruitment, screening or conduct of the FROG feasibility trial (Part 1) are eligible to take part in the embedded mixed methods perspectives feasibility study with no exclusions.

### Part 2 Method: Focus groups and interviews healthcare professionals

***Recruitment and sampling***

Social media advertising and email invitations through the Paediatric Emergency Research in the United Kingdom and Ireland (PERUKI) and General and Adolescent Paediatric Research Collaborative in the United Kingdom and Ireland (GAPRUKI research networks will be used to invite UK HCPs to attend one of up to five online focus groups (mix of healthcare practitioners, approximately 6 to 8 in each group). For healthcare practitioners unable to attend the focus group, up to ten telephone interviews will be conducted. A researcher will send interested HCPs a Participant Information Sheet and provide an opportunity for questions. If they would like to participate, a link to an online consent form will be sent for completion prior to the focus group or interview as well as a list of potential outcomes to read before the focus group or interview.

***Focus group and interview procedures***

Focus groups, interviews and topic guides will be informed by early parent/child interview findings to further explore study acceptability, feasibility, and design, including prioritised outcome measures. Clinical scenarios (vignettes) will be presented to elicit views on optimal methods of urine collection by population and suitability for recruitment to a future study. Consent for audio recording of interviews will be checked verbally before the focus group or interview begins.

### Data analysis part 2 mixed methods perspectives study of feasibility

Interviews and focus groups will be transcribed, checked and anonymised as the study progresses. QSR NVivo software will be used to assist in the organisation and indexing of qualitative data. Whilst reflexive thematic analysis ^41^ will be informed by the constant comparison approach, the focus will be modified to fit with the criterion of catalytic validity, whereby findings should be relevant to future research and practice (in particular, the design of the definitive RCT). Quantitative data from parent questionnaires will be analysed using SPSS software, and descriptive statistics and exact tests will be used, as appropriate. Data from each method will be analysed separately then synthesised through constant comparative analysis to assess Part 2 objectives using the Adapted Framework of Acceptability ^42^.

## Part 3 Consensus meeting

The final phase of the study will involve a face-to-face consensus meeting bringing together stakeholders from PERUKI, GAPRUKI, PPI (e.g. PPI members, parents from Parts 1 and 2/), medical and nursing staff from general practice, ED, inpatient and outpatient settings. The aim is to bring together key stakeholders to review all the data and seek consensus on whether or not a trial is feasible and acceptable to conduct. If it is deemed feasible, consensus will be sought on a non-invasive sampling arm, and one or two invasive sampling arms (TUBC and/or SPA) for use in a future comparative study.

### Inclusion criteria

Parents/guardians of children (0 to under 16 years) and children (aged 7 to under 16 years) who are approached to participate in Part 1, including those who decline randomisation will be included. Parents/guardians of children (0 to under 16 years) and children (aged 7 to under 16 years) who have required urine testing in hospital setting for suspected UTI in the last three years are also eligible to take part in Part 3 of the study. Healthcare practitioners (doctors, nurses, research staff and Allied Health Professionals) involved or not involved in recruitment, screening or conduct to the FROG feasibility trial (Part 1) are eligible to take part in the consensus meeting.

### Exclusion criteria

Those who present with language issues (not overcome with use of translators and available translated information sheets) and those who decline consent will be excluded to take part in Part 3 of the study.

### Part 3: Consensus meeting method

A matrix of 40 key stakeholders will be developed. This will include participants involved in Part 1 and Part 2 who registered their interest in participating in the consensus meeting, in addition to co-investigators, advisory group contacts and subject matter experts from literature review searches. Purposeful sampling will be undertaken across fields of expertise and patient groups to help ensure the meeting attendees are representative of key stakeholder groups. This will involve an email invitation and parents/PPI partners who attend will be compensated for their time. Informed consent will be sought from each participant before the meeting begins with an opportunity for questions. Each aspect of the study including overall acceptability, design, interventions, population of inclusion and outcomes will be discussed. Any areas of disagreement and study feasibility will be discussed, with consensus opinion of relevant stakeholders on key preferred scenarios sought. A voting system (e.g. Turning Point) will be used to help establish consensus if needed. At this stage, if deemed feasible, elements of a definitive trial will be determined.

## Discussion

Timely identification and management of urinary tract infections (UTIs) in infants, children, and young people is essential to prevent adverse outcomes, including renal scarring and long-term kidney damage ^10^. While midstream urine samples are preferred, many children are unable to provide them necessitating alternative sampling methods. Non-invasive techniques such as clean catch urine (CCU) are widely used in the UK but are associated with high contamination rates and practical challenges ^9– 12^. In contrast, invasive methods such as transurethral bladder catheterisation (TUBC) and suprapubic aspiration (SPA) yield cleaner samples ^9^, yet their acceptability and feasibility in routine UK practice remain underexplored.

International guidelines often favour invasive sampling due to lower contamination rates ^14– 18^, and these procedures are considered low-risk when performed by trained professionals ^9– 12, 23, 33^. However, historical concerns about emotional and physical trauma ^19, 21, 22^ and limited UK-based evidence on parent and healthcare professional (HCP) perspectives ^23^ highlight the need for a child-centred, context-specific approach. The FROG feasibility study will address these gaps by evaluating the acceptability and practicality of both invasive and non-invasive urine sampling methods in outpatient, emergency, and clinic settings across the UK.

Using mixed methods and stakeholder involvement, the FROG study will assess recruitment processes, sampling acceptability, and trial design feasibility. Upon completion, progression to a definitive randomised controlled trial (RCT) conducted in the UK will be guided by the following criteria:

(a) Willingness and ability of HCPs to screen and recruit eligible children during Part 1 of the study. This will be demonstrated by recruitment of at least 33% of eligible children.

(b) Mixed methods data on willingness to screen and recruit patients, from Part 2.

(c) Acceptability, or not, of the definitive study, including the invasive urine sampling intervention to parents/guardians, to health care professionals as evidenced by Part 2 data (mapped to the Theoretical Framework of Acceptability) and Part 3 consensus data, as well as expressions of interest for the definitive study.

(d) Development of recruitment and consenting procedures, with associated information materials, that are acceptable to children/parents/guardians based on qualitative insight from families in Part 2.

(e) Selection of suitable patient-centred primary and secondary outcomes through consensus in Part 3, resulting in a study design that addresses a clinically meaningful research question with adequate power.

(f) Evidence of an adequate number of eligible children to deliver the proposed definitive RCT within a reasonable timeframe.

The FROG study will generate essential data to inform the design and implementation of a definitive trial, addressing a critical gap in paediatric UTI management in the UK.

## Declarations

### Ethics approval

The FROG trial received ethical approval from the North East - Newcastle & North Tyneside 1 Research Ethics Committee on 13 ^th^ February 2025. Reference 24/NE/0222.

## Abbreviations

**Table:** 

Acronym	Full Wording
AE	Adverse Event
CCU	Clean Catch Urine sample
CI	Chief Investigator
CONSORT	Consolidated Standards of Reporting Trials
CRF	Case Report Form
CTA	Clinical Trial Authorisation
CTIMP	Clinical Trial of an Investigational Medicinal Product
CTU	Clinical Trials Unit
DMEC	Data Monitoring and Ethics Committee
DMP	Data Management Plan
ED	Emergency Department
FROG	Feasibility of Randomising to invasive and non-invasive urine samplinG
GAPRUKI	General and Adolescent Paediatric Research Collaborative in the United Kingdom and Ireland
GCP	Good Clinical Practice
GP	General Practitioner
HTA	Health Technology Assessment
HRA	Health Research Authority
IB	Investigator’s Brochure
ICH	International Conference of Harmonisation
ISF	Investigator Site File
ISRCTN	International Standard Randomised Controlled Trial Number
ITT	Intention to Treat
MHRA	Medicine and Healthcare Products Regulatory Agency
ModRUM	Modular Resource Use Measure
NHS	National Health Service
NICE	National Institute for Health and Care Excellence
NIHR	National Institute for Health Research
PERUKI	Paediatric Emergency Research in the United Kingdom and Ireland
PIS	Patient Information Sheet
PPIE	Patient and Public Involvement and Engagement
RCT	Randomised Controlled Trial
REC	Research Ethics Committee
SPA	Suprapubic Aspiration
SUDS	Subjective Units of Distress Scale
TUBC	Transurethral Bladder Catheterisation

## Data Availability

No data is associated with this article as this is a study protocol. The statistical analysis plan will be made available on reasonable request upon email to t.waterfield@qub.ac.uk. The Standard Protocol Items: Recommendations for Interventional Trials (SPIRIT) checklist may be accessed https://doi.org/10.17605/OSF.IO/HYDNU ^43^.
