# Supplementary material for: Determining the feasibility of randomising infants, children and young people to invasive and non-invasive urine sampling techniques (FROG): Protocol for a multicentre randomised controlled feasibility trial and mixed methods perspectives’ study of RCT feasibility
Source: NIHR Open Res. 2025 Nov 21;5:108. [Version 1] doi: 10.3310/nihropenres.14114.1 (PMC12741557; doi:10.3310/nihropenres.14114.1)
Supplement: Peer review history

# Reviewer report: Reviewer response for version 1

**DOI:** 10.3310/nihropenres.15350.r38670

**Published:** 2025-12-26

**Authors:** Naqash Sethi

**Affiliations:** 1 Department of Paediatric and Adolescent Medicine, Rigshospitalet (Ringgold ID: 53146), Copenhagen, Capital Region of Denmark, Denmark; 2 Faculty of Health and Medical Sciences, University of Copenhagen, Copenhagen, Denmark

**Review of NIHR Open Research 2025, 5:108**

**Title:** *Determining the feasibility of randomising infants, children and young people to invasive and non-invasive urine sampling techniques (FROG): Protocol for a multicentre randomised controlled feasibility trial and mixed methods perspectives study of RCT feasibility* by Miller PT et al.

 The proposed feasibility trial, parent and patient interview study, and stakeholder consensus meeting investigating how to design a definitive randomized controlled trial comparing invasive versus non-invasive urine sampling in infants, children, and young people is highly relevant and much needed. As highlighted in the manuscript, guidelines currently differ regarding when to recommend invasive versus non-invasive urine sampling, with each approach having important advantages and disadvantages. Accordingly, a randomized controlled trial addressing this topic, with inclusion of both patient- and parent-centered outcomes, is warranted.

**COMMENTS**

**1. Recommended urine collection method in other guidelines**

 In the introduction, the authors correctly state that European and North American guidelines generally favor invasive sampling methods. However, no age range is provided. These guidelines primarily recommend invasive urine sampling in **non–toilet-trained children**, whereas **clean-catch sampling is recommended in children with established bladder control**. I recommend including this age-related clarification in the introduction.

**2. Appropriate population of interest **The proposed trial plans to compare invasive versus non-invasive urine sampling in all pediatric patients under 16 years who are not toilet trained (as stated in Work Package 1 in Figure 2).

- European and North American guidelines generally only recommend invasive urine sampling in non–toilet-trained children, typically below 2–3 years of age. In older children, invasive collection is both technically difficult and may require restraint or sedation, which raises feasibility and ethical concerns. Therefore, I find it challenging—and potentially ethically problematic—to include children older than approximately 2–3 years in a study where 50% would be randomized to invasive sampling.  **I strongly recommend limiting inclusion to non–toilet-trained children under three years of age**, randomized to initial invasive versus initial non-invasive sampling.
- Under the inclusion criteria, “Cannot provide a mid-stream urine sample (are not toilet trained)” may risk misinterpretation, as some non–toilet-trained children may still be able to provide a midstream sample. I recommend revising this to: **“Not toilet trained.”**

**3. Definition of contamination**

 In Part 1 of the Methods, Secondary Outcome #7 is “Rates of contamination by urine collection method.” Please provide a clear definition of contamination and describe how this will be assessed and adjudicated.

**4.** **Pain score**

 In Part 1 of the Methods, Secondary Outcome #10 is “Pain score associated with urine sampling.” Please clarify how pain will be measured in both verbal and nonverbal children. This is later mentioned in the Data Collection section, but I recommend either listing the pain assessment tools in the outcomes section or explicitly referring to the Data Collection section here.

**5. Figure 6**

 In Figure 6, antibiotic administration is shown at “baseline approx. 1 hour,” which appears to precede urine sample collection (“approx. 2–4 hours”). I believe this may be an error. Antibiotic therapy should generally be initiated **after** urine collection, except in rare acute circumstances where treatment cannot be delayed. I recommend revising Figure 6 to avoid misinterpretation.

Is the study design appropriate for the research question?

Yes

Is the rationale for, and objectives of, the study clearly described?

Yes

Are sufficient details of the methods provided to allow replication by others?

Yes

Are the datasets clearly presented in a useable and accessible format?

Yes

Reviewer Expertise:

Urinary tract infection, Evidence-based medicine, Implementation research, 'Choosing wisely'

I confirm that I have read this submission and believe that I have an appropriate level of expertise to confirm that it is of an acceptable scientific standard, however I have significant reservations, as outlined above.

# Reviewer report: Reviewer response for version 1

**DOI:** 10.3310/nihropenres.15350.r38525

**Published:** 2025-12-26

**Authors:** Ioana-Cristina Olariu

**Affiliations:** 1 University of Medicine and Pharmacy,, Timisoara, Romania

The study protocol "Determining the feasibility of randomising infants, children and young people to invasive and non-invasive urine sampling techniques (FROG): Protocol for a multicentre randomised controlled feasibility trial and mixed methods perspectives’ study of RCT feasibility" designed by the authors refers to the collection of urine samples in children under 16 years old if there is suspicion of a urinary tract infection.

 Urine samples can be collected invasively or non-invasively, with invasive methods having a much lower risk of sample contamination. However, non-invasive methods are preferred, as they are less stressful for both the child and the parent/guardian and do not require a specially trained medical practitioner. Therefore, it is very unlikely that parents/guardians or children will easily accept suprapubic aspiration, so statistically the results may not be the most adequate. The study protocol does not mention the risks of infection from invasive procedures.

 It is not clear how information about the study can be disseminated through social media.

 Some clarifications are needed for each first abbreviation in the text.

Is the study design appropriate for the research question?

Yes

Is the rationale for, and objectives of, the study clearly described?

Yes

Are sufficient details of the methods provided to allow replication by others?

Yes

Are the datasets clearly presented in a useable and accessible format?

Yes

Reviewer Expertise:

Pediatrics

I confirm that I have read this submission and believe that I have an appropriate level of expertise to confirm that it is of an acceptable scientific standard.

# Reviewer report: Reviewer response for version 1

**DOI:** 10.3310/nihropenres.15350.r38529

**Published:** 2025-12-16

**Authors:** jharna mandal

**Affiliations:** 1 Jawaharlal Institute of Postgraduate Medical Education and Research (JIPMER),, Puducherry, India

The study focuses on an important aspect of diagnosing UTI in children- Sample collection. Albeit, if the right sample is collected in the right manner, the results will be right. The study design is robust, well planned and clearly mentioned. 

 Please clarify if the group will be exposed to any training module regarding the sampling, and what the pitfalls of a poorly collected specimen are and the benefits of having a well-collected specimen submitted for culture. This can be performed/introduced at some point of the study, once the opinion of the parents/PPI is sought.

 This will spread awareness and will ease the apprehensions of the parents/Guardians of the children. Counselling of caretakers is an important aspect of the well-being of the patient, here the child. Healthcare providers must insist on this. For laboratory diagnosis of culture, a detailed plan of sample processing and culture interpretation should be a part.

 Questions should be addressed: How will they address mixed growths in culture? A definition of a contaminated specimen should be available. How many repeat samples are being requested for each patient? What is the reliability of microscopic examination of urine samples? The dipstick method will help rule out UTI and not rule in, hence, such tests should not be included in the protocol.

Is the study design appropriate for the research question?

Yes

Is the rationale for, and objectives of, the study clearly described?

Yes

Are sufficient details of the methods provided to allow replication by others?

Yes

Are the datasets clearly presented in a useable and accessible format?

Not applicable

Reviewer Expertise:

Clinical Microbiology, Infection prevention, Control and antimicrobial stewardship.

I confirm that I have read this submission and believe that I have an appropriate level of expertise to confirm that it is of an acceptable scientific standard.
